# Script-driven imagery of socially salient autobiographical memories in major depressive disorder

**DOI:** 10.1038/s41598-023-41486-7

**Published:** 2023-09-04

**Authors:** Julia Gillard, Aliza Werner-Seidler, Tim Dalgleish, Jason Stretton

**Affiliations:** 1 Anglia Ruskin University, Cambridge, UK; 2 Black Dog Institute, University of New South Wales, Sydney, Australia; 3 Cambridgeshire and Peterborough NHS Foundation Trust, Cambridge, UK; 4 Medical Research Council Cognition and Brain Sciences Unit, University of Cambridge, 15 Chaucer Road, Cambridge, CB2 7EF UK

**Keywords:** Social neuroscience, Neuroscience, Emotion, Insula, Human behaviour

## Abstract

Cues of social rejection and affiliation represent proximal risk and protective factors in the onset and maintenance of depression. Such cues are thought to activate an evolutionarily primed neuro-cognitive alarm system, alerting the agent to the benefits of inclusion or the risk of social exclusion within social hierarchies focused on ensuring continued access to resources. In tandem, autobiographical memory is thought to be over-general and negatively biased in Major Depressive Disorder (MDD) which can contribute to maintenance and relapse. How memories of social rejection and affiliation are experienced and processed in MDD remains unexplored. Eighteen participants with recurrent and chronic MDD and 18 never-depressed controls listened to and vividly revisited autobiographical social experiences in an ecologically valid script-driven imagery paradigm using naturalistic memory narratives in an fMRI paradigm. Memories of Social Inclusion and Social Rejection broadly activated a common network of regions including the bilateral insula, thalamus and pre/postcentral gyrus across both groups. However, having a diagnosis of MDD was associated with an increased activation of the right middle frontal gyrus irrespective of memory type. Changes in positive affect were associated with activity in the dorsal ACC in the MDD group and in the insular cortex of the Control group. Our findings add to the evidence for complex representations for both positive and negative social signals in MDD and suggest neural sensitivity in MDD towards *any* socially salient information as opposed to selective sensitivity towards negative social experiences.

## Introduction

The ability to detect and respond to diverse signals of social inclusion and exclusion is critical to the establishment and maintenance of relationships, groups and social hierarchies^1^. Formation of these social attachments affects aspects of our narrative selves, motivations, goals, behaviours, self-identity and mental health^2^ and is in part shaped by access to the vast repository of autobiographical memories concerning social discourse. Major Depressive Disorder (MDD) is associated with maladaptive processes in each of these domains; hypersensitivity to social cues of rejection and affiliation^3^; a propensity to overgeneralise negative autobiographical memories (AM); and an inability to access more specific positive memories^4^. Little is known of the interaction between these processes and how they may contribute to the maintenance of low mood and maladaptive social functioning observed in MDD.

The consequences of social rejection are important in the clinical context of MDD, with individual differences in interpersonal rejection sensitivity found to mediate the relationship between early adverse life events and depressive symptoms in adult life^5^, suggesting that memories of such events can have lasting impacts. Given the fundamental human need to belong and the relationship between social connectedness and well-being^1^, evolutionary theoretical frameworks such as Sociometer Theory^6^ seek to elucidate mechanisms for the modification and adaptation of social strategies and behaviours in response to social feedback. Heightened sensitivity to social rejection cues and processing of these cues feeds into state levels of self-esteem, which thus serves as a gauge of interpersonal relationship status^1^. The Social Risk Hypothesis (SRH) of depressed mood^3,7^ extends this notion to propose that heightened sensitivity to social cues is a cardinal feature of depressed mood. It argues that depressed individuals adopt a risk-averse approach to social interactions with the overall aim of reducing the threat of social exclusion^3^, engaging in social withdrawal and/or signalling submissiveness and subordination to conspecifics to avoid defeat, or to elicit assistance^3,8^. In the long-term, this risk-averse state maintains low mood and may contribute to other maladaptive social functions^9–11^. Consequently, the SRH posits easier access to memories of past failures, relative to past successes, as a maintenance factor of MDD and a risk factor for future depressive relapse^3^.

At the neural level, monitoring socially evaluative signals of others has generally been associated with activation of a network of regions including the subgenual anterior cingulate cortex (sgACC^12^), anterior insula (AI^13^), and ventral striatum (VS^14^) with negative social evaluations also associated with activity in the lateral prefrontal and orbitofrontal cortex (OFC^15^). More specifically, meta-analyses of personal experiences of social pain and rejection suggest both dorsal and ventral Anterior Cingulate Cortex (ACC) are associated with social pain elicitation and subjective distress^16^ and the broader involvement of the dorsal ACC (dACC) and AI in response to social rejection^17^ as well as socially affiliative signals^18^. Together these regions constitute an affective salience network involved in monitoring socially evaluative threat to the self^19^. If there is indeed heightened reactivity to social cues of rejection and inclusion in regions of the affective salience network, this may represent a neural mechanism for the broader modification and adaptation of social behaviour, as proposed by models such as Sociometer Theory^6,20^ and the SRH^3^.

The SRH posits access to past failures would be more easily accessible than past successes^3^. The present study therefore attempts to control the confound of accessibility inherent to depressed participants by pre-generating autobiographical memories of both social rejection and social inclusion and then relaying the same memories back to participants under fMRI conditions. At both the behavioural (affective) and neural level, we used ecologically valid naturalistic stimuli—self-generated autobiographical memories of social rejection and inclusion—in individuals with a diagnosis of recurrent, chronic MDD, compared to participants who have never experienced depression. Such memories represent a vast repository for salient naturalistic social experiences and emotions that can be drawn upon to inform, establish or maintain our current social narrative and interpersonal relationships and consequently, our emotional state. The recollection of these emotionally salient autobiographical memories can provide an ecologically valid measure of subjective distress and pleasure in response to social rejection and inclusion and provide insight into the underlying neural processing of social signals in depressed compared to healthy individuals. Secondly, using a memory-based paradigm is ethically preferable to a real-time rejection based paradigm for individuals currently in a depressive episode. We first hypothesised that all participants would show a common neural substrate associated with regions of the affective salience network to memories of *both* social rejection and social inclusion in line with previous work^18^. Secondly, we hypothesised that individuals with MDD would show heightened activity in regions of the affective salience network relative to never-depressed controls reflecting greater sensitivity to social affiliative/threat signals as predicted by the SRH^3^. Finally, we hypothesised that activity in affective salience regions would be associated with increases in both positive and negative affect in depression.

## Materials and methods

### Participants

Eighteen participants experiencing a current Major Depressive Episode and meeting criteria for a diagnosis of recurrent Major Depressive Disorder (MDD; 13 female, 5 male; 34.11 ± 10.9 years) on the Structured Clinical Interview for the DSM-IV^21^, and 21 healthy controls who had never met criteria for MDD (10 female, 11 male; 35.30 ± 16.1 years) were recruited from volunteer panels at the MRC Cognition and Brain Sciences Unit, University of Cambridge. Participants were right-handed, with no history of brain injury, normal or corrected-to-normal vision and no hearing impairments.

### Tasks and procedure

All participants completed two research sessions on separate days, a Memory Generation Session and a Neuroimaging Session (see below). The study was carried out in accordance with the Declaration of Helsinki and Good Clinical Practice and approved by the Cambridge Psychology Research Ethics Committee (PRE.2014.43). All participants provided written informed consent and received monetary compensation.

### Memory generation session

In the initial Memory Generation session, participants provided detailed narratives of 18 autobiographical memories (six social rejection memories, six social inclusion memories, and six emotionally neutral social memories; e.g., *shopping in the presence of other individuals*). For each memory, participants were asked to emphasize sensory details and visceral reactions as there is evidence that accessing these features enhances the emotive impact of the memories^22,23^. Participants rated the vividness and intensity of their memories, and four subjective components describing how the memory makes them feel when recollecting it now (distress, rejection, inclusion, positivity), on Likert scales ranging from 0 (“Not at all”) to 10 (“Extremely”). These four subjective affect ratings were compiled into a negative mood index (NMI; the average of the distress and rejection ratings), and a positive mood index (PMI; the average of the inclusion and positivity ratings). The NMI was then subtracted from the PMI, resulting in an overall composite mood measure of affect that could range from − 10 (very negative/rejected) to + 10 (very included/positive). Participants were additionally asked to describe how the memory made them feel at the time of the original experience, using the same four subjective mood states as above, to ensure comparability across groups in terms of the affective impact of the original experiences.

The 18 memory narratives were then edited into 30-s audio-scripts narrated in the first person, present tense and audio-recorded by a research assistant sex-matched to the participant, to be used as stimuli in the fMRI paradigm. While depression may be characterized by memory biases, including reduced specificity and facilitated retrieval of negative over positive experiences, recalling autobiographical memories using a script-driven imagery approach can powerfully re-elicit salient emotions experienced in the present in spite of these biases^22,23^. In line with previous research using cognitive manipulations to facilitate the recall of specific autobiographical memories, including in depressed participants^24–26^, the present paradigm thus falls in line with previous studies^22,23,27^. In this initial session, participants were also assessed with the mood module of the SCID-I^21^ and completed the Beck Depression Inventory (BDI-II), assessing current and residual symptoms of depression^28^. See Supplementary Methods for more information on these clinical measures.

### Neuroimaging session

**Figure 1:**
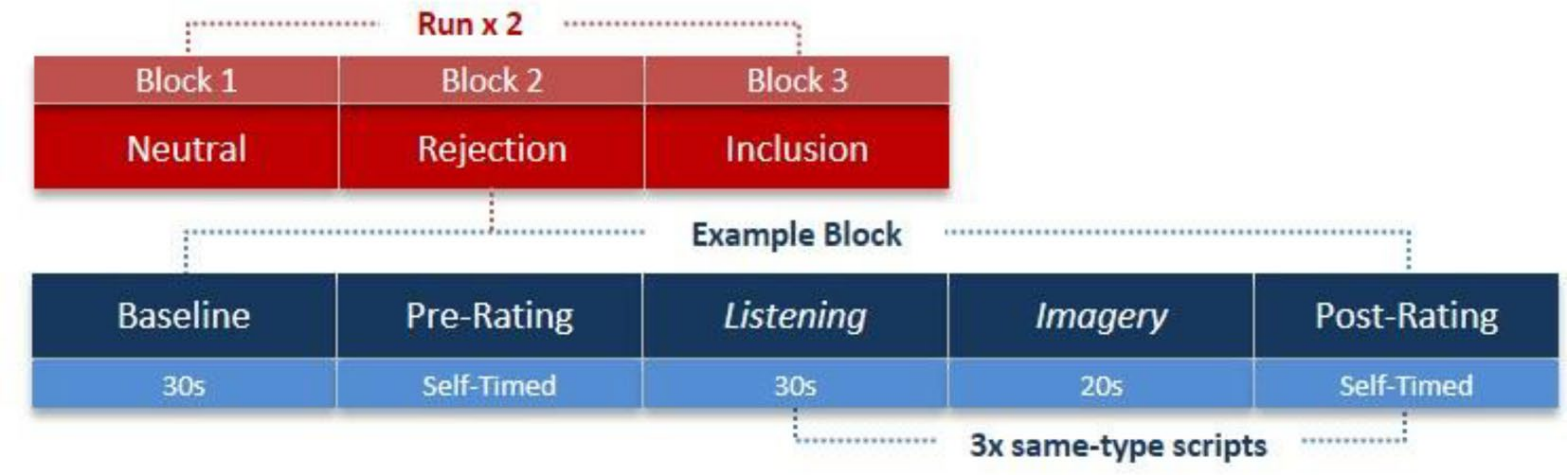
Script-Driven Imagery Paradigm adapted for fMRI.

The subsequent Neuroimaging Session took place on a separate day, approximately one week later. The scanning paradigm used a block-design comprising two functional runs with three blocks (neutral, rejection, inclusion memory scripts) per run presented in fixed order so that participants did not end the session with the aversive memory (Fig. 1). The fMRI paradigm presented three 30-s audio memory scripts in each of the three blocks (neutral, rejection and inclusion memories; nine memories in total) during which participants were instructed to close their eyes and immerse themselves in each memory. Each memory was followed by a 20-s silent imagery period^23^ in which participants were encouraged to elaborate on the contents and emotional salience of the memory they had just heard described. A tone cue followed each silent imagery period to indicate to participants to open their eyes. Pre- and Post- affect ratings of current mood were recorded in the scanner directly before and after each memory + silent imagery epoch. Participants also rated their mood prior to each block of three same-valence memories following a 30-s closed-eye baseline period. Mood ratings comprised levels of current subjective distress, rejection, inclusion and positivity in response to the memories on the same 11-point Likert scale as in the Memory Generation session. Again, the four subjective mood states were compiled into a NMI (the reverse scored average of the distress and rejection ratings), and a PMI (the average of the inclusion and positivity ratings) to be entered into subsequent analyses. The composite mood index (PMI-NMI) was used to compare elicited affect between the neuroimaging session and the memory generation session. An additional 30-s washout clip depicting an ocean sunset was presented between the rejection and inclusion memory blocks to help return affect to baseline (scores on neutral memories). The order of individual scripts within each block was randomized. The total approximate duration was 25 min per run.

### Stimulus presentation within the scanner

Auditory presentation of the stimuli inside the scanner was delivered via Sensimetric’s S14 headphones following the application of a custom equalization filter (© 2010 Sensimetric Corporation—www.sens.com, version 2.1) in combination with ear defenders to attenuate scanner noise. The headphones were connected via a desktop PC running MATLAB R2013a (Mathworks; Sherbon, Massachusetts) and presented using the psychophysics toolbox. Simultaneously, participants were asked to provide affect ratings using a button box. Visual presentation was provided via a custom-built mirror stereoscope, with the participant’s head stabilized by a chin-and-head rest. The effective viewing distance was 50 cm with a resolution of 1024 × 768 and a visual angle of 16.7 degrees.

### Data acquisition

A 3 T Siemens Tim Trio MRI scanner with a 32-channel head coil was used to acquire a structural T1-weighted MPRAGE image (1-mm isotropic voxels) and functional data of ~ 600 whole-brain T2*-weighted EPI volumes with 32 oblique axial slices that were 3.5 mm thick, and an in-plane 64 × 64 matrix with resolution of 3 × 3 mm, TR 2 s, and TE 30 ms (two runs). FMRI data were pre-processed and analysed using MATLAB and SPM12 (Statistical Parametric Mapping Software; Wellcome Centre for Imaging Neuroscience, London, UK; http://www.fil.ion.ucl.ac.uk/spm/software/spm12/).

### Statistical analysis

#### fMRI pre-processing

Raw DICOM images were converted to NIFTI format, realigned within and across both runs to correct for motion and then each of the images was matched/resliced to the first image of the time series and a mean of these aligned images was generated. The mean BOLD image was co-registered with the T1 image, segmented and spatially normalized to MNI template space. The resulting warps were applied to all volumes, with a 3 mm isotropic voxel interpolation, followed by a 3D 8 mm isotropic Gaussian kernel smoothing.

#### fMRI analyses

Our analytic approach followed current guidance for clinical neuroimaging studies^29^. For fMRI analysis we ran a two-level random effects analysis using SPM12. At the first level, a linear convolution GLM was applied to the time series within each voxel across both runs. For each run, the BOLD response was modelled by convolving a canonical hemodynamic response function (HRF) to each memory type (neutral, rejection, inclusion) during listening, silent imagery, closed-eye baseline, baseline ratings, post-script ratings, washout movie clip, and text instructions, as well as six additional regressors generated per run to account for rigid-body movement (realignment parameters). Silent Imagery contrasts were generated for each memory type relative to baseline and subtraction contrasts were generated for Social Inclusion and Social Rejection versus Socially Neutral memories respectively. These subtraction contrasts were taken to the second-level and entered into a full factorial ANCOVA with Group (2 levels: controls, depressed) and Memory Type (2 levels: Social Inclusion, Social Rejection) as the conditions of interest. We corrected for multiple comparisons using a cluster-wise random field theory (_*pRFT-FWE*_) threshold of 0.05, using an initial cluster-defining height threshold of *p* < 0.001 uncorrected and a cluster extent of *k*_*e*_ = 202 (https://doi.org/10.5281/zenodo.1689890). The same contrasts and analysis was conducted for Active Listening and can be found in the Supplemental Results. Significant main effects of Group from the ANCOVA output were followed up to examine our a priori hypothesis that the MDD group would exhibit greater activation relative to the healthy control group. Appropriate follow up analysis of any significant main effects of Memory Type or Group × Memory Type interactions were also planned. All fMRI analyses included sex as a co-variate to address the sex imbalance across groups (control group has 50% female and depressed group has 72% female). Despite a non-significant chi-square finding, this approach ensured that any sex differences would not mask differences between Healthy Controls and MDD groups.

Finally, we wanted to explore the critical relationship between task-related neural activity during Silent Imagery and changes in positive and negative affect experienced after listening to each memory. We thus entered the PMI and NMI scores as covariates of interest in a full factorial ANCOVA with a between-subject factor of group (2 levels; MDD and Controls) and a within-subject factor of memory type relative to baseline (3 levels; Inclusion, Rejection and Neutral) in order to improve sensitivity to overall changes in affect. We set the mood indices to interact with the group factor allowing generation of contrasts for each group’s PMI and NMI respectively, regressed against activity across all memory types. For completeness, we also examined PMI and NMI across all participants for respective inclusion and rejection memories.

## Results

### Participants

Three healthy control participants were excluded from the final analysis due to acquisition difficulties in the scanner. Demographic data for the remaining 18 healthy control participants and 18 participants in the MDD group are presented in Table 1. The groups were well-matched with the anticipated exception of self-reported depression symptoms measured with the BDI. For information on co-morbidities and medication see Table 2. While chi-squared analyses revealed no significant association between group and sex, the limited sample size and numerical sex imbalance indicated that sex be nonetheless included as a co-variate in our analyses.

**Table 1:** Demographic Characteristics. Numbers are *n*s unless otherwise stated.

	Controls n = 18	Depressed n = 18	Total N = 36	t/X^2^	*P*
Sex					
Male	9	5	14	1.87	0.17
Female	9	13	22		
Age, years					
Mean	35.28	34.11	34.69	0.25	0.81
SD	16.95	10.92	14.07		
BDI-II					
Mean	10.11	22.33		** − **4.79	< .001
SD	4.43	9.89			
National adult reading test					
Mean	9.33	7.78	8.56	0.81	0.42
SD	5.99	5.48	5.71		
Ethnicity					
Caucasian	17	16	33	0.36^a^	0.55
Other	1	2	3		
Marital status					
Single/unmarried	14	8	22	6.75^a^	0.15
Married	2	7	9		
Separated/divorced	1	2	3		
Other	1	1	2		
Education					
Completed HSC/Yr 12	8	8	16	5.07^a^	0.54
Other*	10	10	20		
Employment status					
Employed	14	13	27	1.54^a^	0.46
Unemployed	4	5	9		
Employment					
Full-time	11	12	23	1.54^a^	0.67
Part-time	5	3	8		
Other	2	3	5		

*BDI-II* Beck Depression Inventory, *HSC* Higher School Certificate.

^a^indicates Fisher’s exact test.

*Other indicates further education after high school.

**Table 2:** Co-morbidities and Medication.

	Controls n = 18	Depressed n = 18	Total N = 36
Co-Morbidities			
Anxiety not otherwise specified	0	3	3
General anxiety disorder	1	0	1
Post-traumatic stress disorder	0	2	2
Social anxiety	0	1	1
Total	1	6	7
Medication			
Citalopram	0	2	2
Fluoxetine	0	3	3
Mirtazapine	0	1	1
Other	0	3	3
Sertraline	0	1	1
Total	0	10	10

Numbers are *n*s unless otherwise stated.

### Affective ratings of memories

The memories generated in the Memory Generation session evoked the anticipated affective reactions as a function of memory type (social inclusion, social rejection, socially neutral) with comparable intensity of response across our two groups, for both emotional impact on recall and for retrospectively rated emotional impact at the time of the original event (see Supplementary Results and Figs. S1–S4).

**Figure 2:**
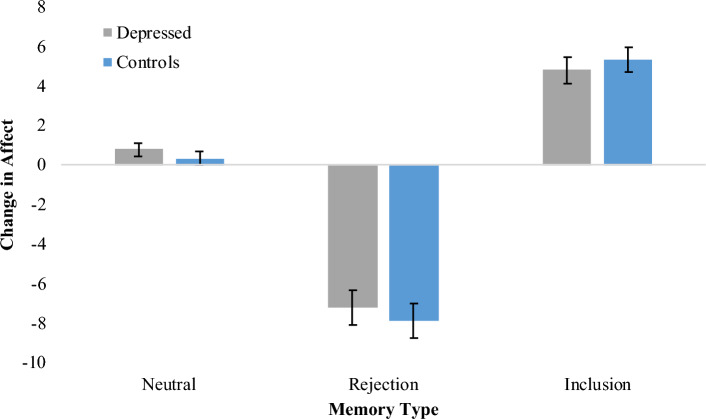
Mean change ± 1SE in composite affect ratings from pre- to post-script processing (lower scores more negative) in response to social memory scripts from the Neuroimaging Session.

We verified the expected differential emotional effects of processing social rejection, inclusion and neutral memory scripts during the Script-induced imagery task in the Neuroimaging Session by comparing the mean changes in composite affective ratings using Analysis of Variance (ANOVA) (see Fig. 2). This revealed a significant main effect of Memory Type (F[1.51,52.91] = 188.91, *p *< 0.001, η_p_^2^ = 0.84). As expected, follow-up t-tests revealed that affect was rated as significantly more negative in response to rejection memories relative to both neutral (t = 13.90, *p *< 0.001, *d* = 2.29) and inclusion memories (t = 15.49, *p *< 0.001, *d* = 2.55), while inclusion memories were rated as highest in positive affect relative to neutral (t = 8.68, *p *< 0.001, *d* = 1.43). Reassuringly, and in line with the data from the initial Memory Generation session, there was no main effect of Group, nor a significant interaction between Memory Type and Group (*F*s < 1). This validates our aim to carefully select and match memories in the initial Memory Generation Session.

### FMRI

**Figure 3:**
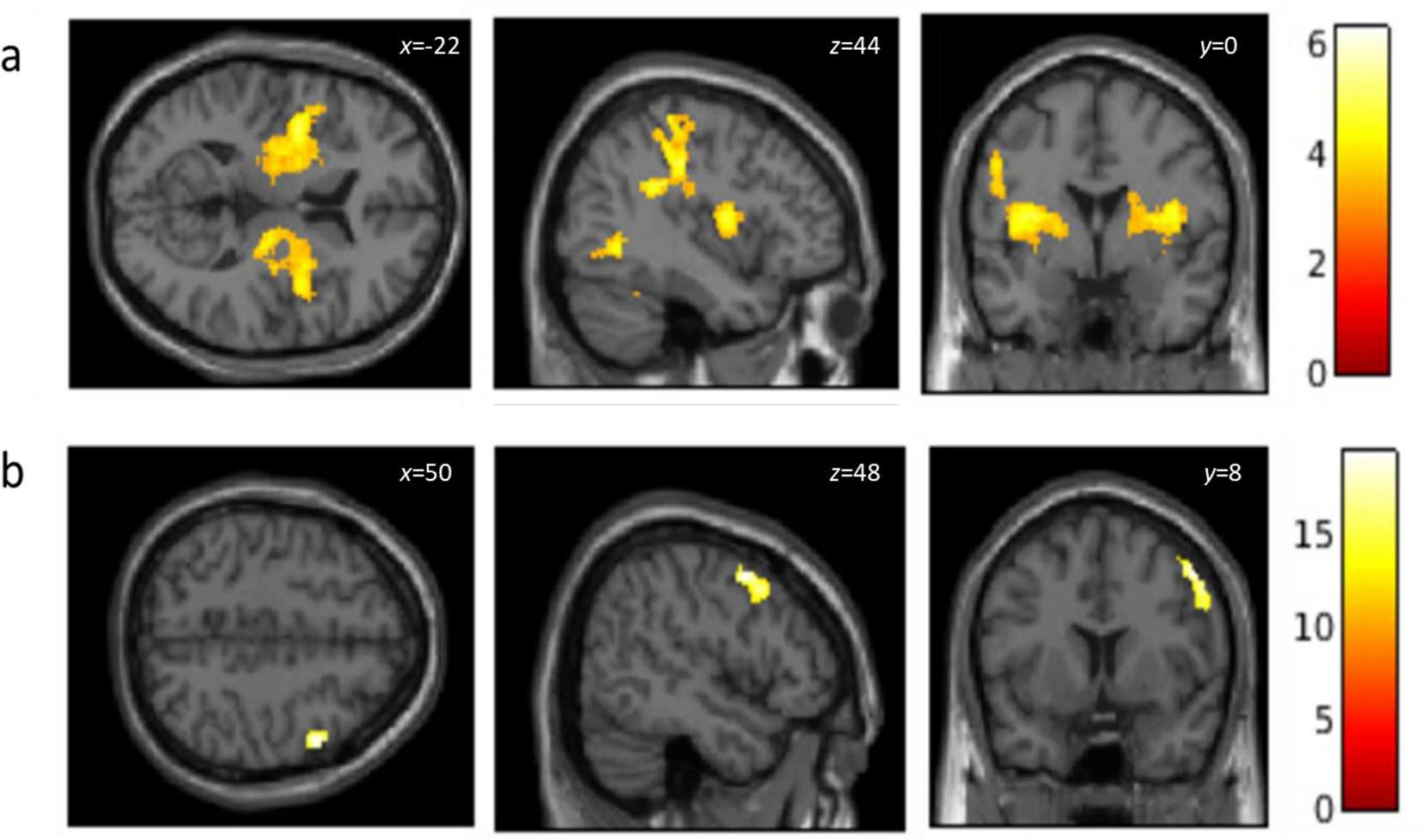
Logical AND conjunction of both Social Inclusion and Social Rejection memories (relative to Socially Neutral memories) during Silent Imagery across all participants (N = 36) revealing activity in the bilateral postcentral gyrus and insula (**a**), and the main effect of Group in the right middle frontal gyrus (**b**). FWE corrected cluster level *p *< 0.05_FWEc_, initial height threshold uncorrected *p* < 0.001 with an extent threshold of k = 202.

Whole brain ANCOVA revealed a positive effect of memory type of both Social Inclusion and Social Rejection memories (relative to Socially Neutral memories) across all participants. Both conditions activated the bilateral postcentral gyrus extending into the bilateral insula, ventral striatum, thalamus and middle and anterior cingulate cortex (ACC) replicating our previous findings^18^ but this time for social autobiographical memories. There was no main effect of memory type, and corroboratively a logical AND conjunction^30^ of both memory types (relative to Socially Neutral) revealed clusters of activity in the bilateral insula, thalamus and pre- and post-central gyri as a common neural substrate for processing social memories of inclusivity and rejection (Table 3 and Fig. 3a). Finally, there was a significant main effect of Group [F (1,67) = 11.85, *p *< 0.05_FWEc_] in the right middle frontal gyrus (MFG) (peak voxel: 50, 8, 48; z = 3.97). Planned comparisons confirmed that irrespective of Memory Type (relative to Socially Neutral), this cluster was more active in the MDD group relative to the Control group (Fig. 3b). There were no significant differences between Inclusion and Rejection memories and there were no significant interactions between Group and Memory Type.

**Table 3:** Peak clusters from a logical AND conjunction associated with Social Inclusion and Social Rejection memories during Silent Imagery across all participants (N = 36).

Anatomical label	*k* voxels	z-value	xyz (mm)
**Right post central gyrus**	**3193**	**5.84**	**40; − 26; 42**
Right parietal operculum		5.52	44; − 22; 28
Right supramarginal gyrus		5.18	48; − 20; 36
**Left post central gyrus**	**4989**	**5.62**	** − 48; − 22; 28**
Left supramarginal gyrus		5.16	** − **48; − 30; 32
Left parietal operculum		5.13	** − **40; − 36; 28
**Left inferior temporal gyrus**	**334**	**5.20**	** − 46; − 60; − 4**
**Right cerebellum exterior**	**1361**	**4.69**	**26; − 72; − 18**
Right fusiform gyrus		4.48	34; − 46; − 24
Right inferior temporal gyrus		4.37	48; − 62; − 4
**Left cerebellum exterior**	**895**	**4.46**	** − 32; − 46; − 24**
Left lingual gyrus		4.37	** − **6; − 72; − 6
Left fusiform gyrus		4.12	** − **24; − 72; − 4
**Left middle occipital gyrus**	**268**	**4.00**	** − 38; − 74; 12**

*p* value; FWE cluster level, initial height threshold uncorrected *p* < 0.001 with an extent threshold of k = 202. Bold font indicates peak co-ordinates of each cluster.

### Relationship with positive and negative mood indices for each group

**Figure 4:**
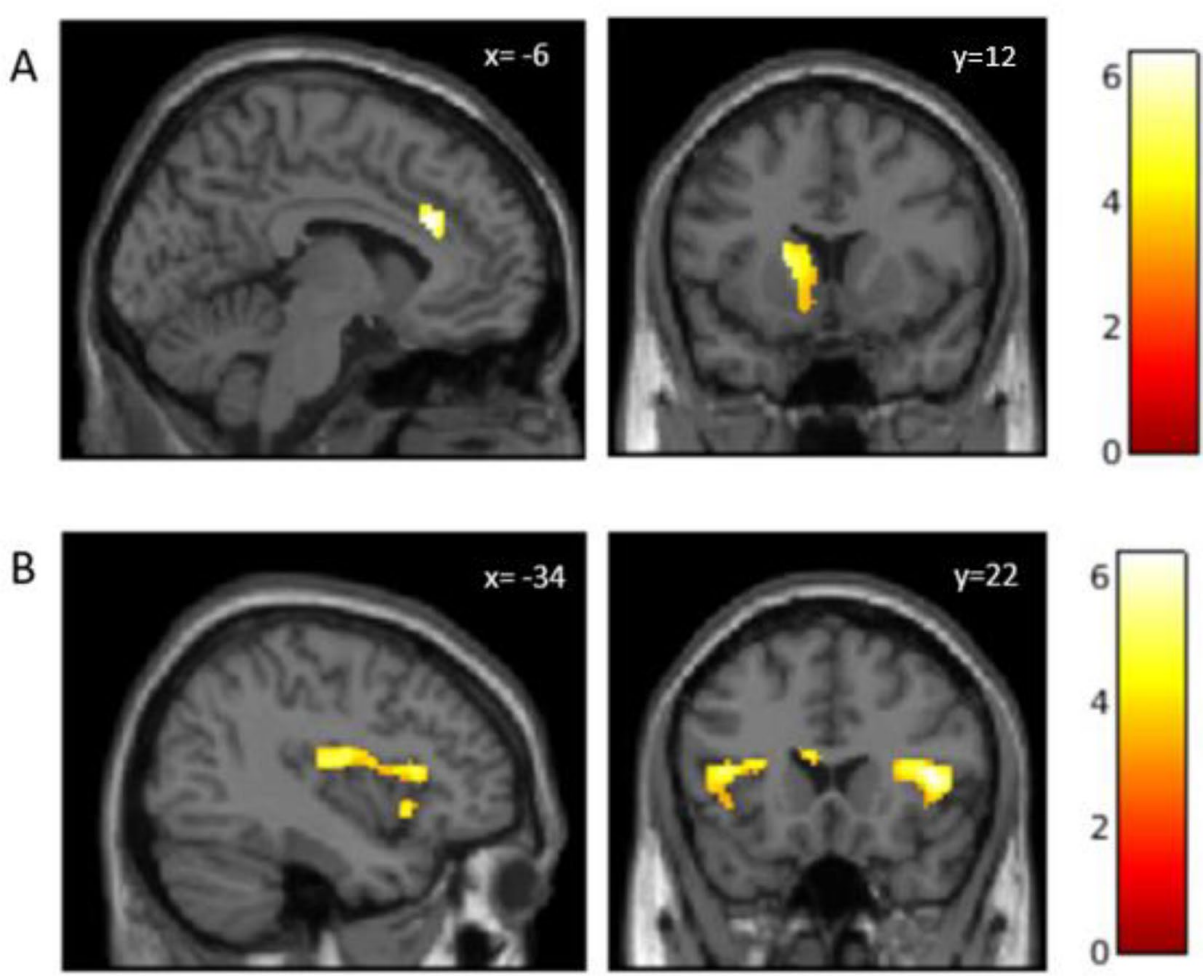
The relationship between activation during Silent Imagery and PMI in the MDD group showing dACC (x = **− **6) and left ventral striatum (y = 12) (**A**) and in the bilateral anterior insula (y = 22) and left posterior insula (x = **− **34) control group (**B**).

In order to explore the relationship between changes in mood following imagery and the related neural activation, we regressed the positive (PMI) and negative (NMI) mood indices against activation for each memory type relative to baseline interacting with group. There was a significant Group × PMI interaction [F (1, 97) = 11.51, *p *< 0.05_FWEc_]. In the MDD group, higher PMI was associated with activation in the left dorsal and ventral striatum (peak voxel =  − 18, 14, 10; z = 5.09) and dorsal Anterior Cingulate Cortex (dACC; peak voxel =  − 6, 26, 24; z = 4.94). In the control group, higher PMI was associated with activation in the bilateral anterior insula (left AI − 34, 14, 12; z = 5.68; right AI 30, 22, 10; z = 4.54) and left posterior insula (− 38, − 16, 16; z = 5.39). There were no areas of activity associated with PMI or NMI across the entire sample for Social Inclusion or Social Rejection memories, respectively (See Fig. 4).

## Discussion

Using a script-driven imagery paradigm, we show that participants with recurrent, chronic MDD are able to retrieve and access specific socially affiliative autobiographical memories which generate positive affect comparable to that of never-depressed controls. In line with our hypothesis, we show common neural processing of socially inclusive and socially rejecting memories relative to socially neutral memories across both groups. Evidence supporting this hypothesis was found in regions including the bilateral insula and cingulate cortex, with increased activity in the right MFG specifically in participants with MDD. Finally, we show that changes in positive, but not negative, affect were related to activation in the dACC and AI in the MDD and control groups, respectively.

These results extend previous work revealing activation across the affective salience network in response to the psychological experience of current social pain^31^ and affiliation^18^ to the domain of autobiographical memory for social inclusion and rejection, and the resulting positive affect associated with it. Further, we show the right middle frontal gyrus, a region typically associated with executive control^32^, shows greater engagement while reimagining memories of social inclusion and rejection in MDD relative to controls. While this region has been shown to be activated within non-social contexts in depression^33^, we speculate that under conditions of social affiliation, this sensitivity may arise from the personal relevance of those social evaluations to one’s current social status^18,34–36^, which may be heightened in depression. These results, strengthened by their ecological validity, contribute to an emerging neuroimaging literature suggesting a dedicated neural substrate for complex representations of both positive and negative social signals^17,18,20,37,38^. While it has hitherto been established that depressed individuals exhibit heightened interpersonal rejection sensitivity and systematic biases in emotional processing at both the behavioural and neural level^39,40^, such prior studies have focused on the experience of negative social experiences, frequently elicited under less ecologically valid conditions. However, the ability to monitor *both* socially inclusive and exclusive salient cues is central to the notion of a more sophisticated index of social processing underscored by our fundamental need to belong^1^. This is supported by empirical evidence that individuals high in interpersonal rejection sensitivity tend to modify their interpersonal behaviour to avoid social exclusion and maintain social acceptance^41^. Finally, our results are in line with wider literature suggesting that social cues involving social acceptance may elicit higher levels and greater intensity of positive affect in depressed individuals, contrary to previous assumptions^36,39,42^. This suggests that depressive symptoms may increase sensitivity to both past and present experiences of both social acceptance.

A key strength of this study was the use of idiosyncratic autobiographical social experiences. This method is a novel and ecologically valid way of assessing social processes in depression. Providing equal footing to both positive and negative self-relevant experiences further reduced the memory biases previously reported in the literature and instead facilitated the elicitation of salient emotions in the present across valences and groups^22,23^. However, future studies investigating the psychological and neural characterization of social experiences would benefit from a more consistent operationalization of intra- and interpersonal contextual factors^43,44^, and the incorporation of non-social affective autobiographical memories to fully account for sociality as the core process driving these common activations.

Our study is limited by a modest sample size which constrained the power to detect group differences and interactions. However, recruiting in-episode participants with MDD is challenging, particularly when trying to recruit more moderate-severe participants with a chronic history. In addition, whilst we also cannot rule out the influence of medication (both current and historic) on the imaging results, it is similarly challenging to recruit medication naïve participants with a chronic history of the condition. Indeed, participants with no history of medication use are unlikely to be representative of the wider population with a chronic pattern of MDD. Finally, the fixed order of presentation could have biased behavioural and neural responses as inclusive memory blocks were fixed to follow rejection memories. However, the washout clip (sunset) showed between these blocks ensured there was no significant difference in baseline mood between blocks, in spite of the potential for a sunset to be positively mood-inducing (see Supplementary Results). Finally, although there was no significant difference in vividness between memory types (see Supplementary Results), we cannot discount the potential effects of strength of recollection vs familiarity during autobiographical memory recall, particularly as insula activation is associated with strength of recollection^45^. Future studies would benefit from collecting ratings of recollection and familiarity.

In sum, we show that script driven imagery of specific, socially salient autobiographical memories recruits a common neural substrate encompassing the affective salience network. Further, we show that regions associated with social pain and rejection are also involved in signals of social affiliation, and relate to the changes in affect experienced as a function of the memories. The findings contribute to an emerging literature suggesting a neural sociometer concerned with the ongoing evaluation of all socially salient information which may be more sensitive in individuals with a chronic history of depressive disorder.

### Supplementary Information

Supplementary Information.

## Data Availability

Due to the sensitive nature of the autobiographical memories used in this study, participants were assured their narratives would remain confidential and would not be shared. However, the fMRI and behavioural data reported in this article will be made available in the CBU Open Data Repository (http://www.mrc-cbu.cam.ac.uk/publications/opendata).
