# Supplementary material for: Script-driven imagery of socially salient autobiographical memories in major depressive disorder
Source: Sci Rep. 2023 Sep 4;13:14577. doi: 10.1038/s41598-023-41486-7 (PMC10477266; doi:10.1038/s41598-023-41486-7)
Supplement: Supplementary file 1 — Supplementary Information. [file 41598_2023_41486_MOESM1_ESM.docx]

# Supplementary Materials

**Title**: Script-Driven Imagery of Socially Salient Autobiographical Memories in Major Depressive Disorder.

**Authors**: Julia Gillard ^a^, Aliza Werner-Seidler ^b^, Tim Dalgleish ^c, d^^, Jason Stretton ^d^^*

**Institution**:

^a^ Anglia Ruskin University, Cambridge, United Kingdom

^b^ Black Dog Institute, University of New South Wales, Sydney, Australia

^c^ Cambridgeshire and Peterborough NHS Foundation Trust

^d^ Medical Research Council Cognition and Brain Sciences Unit, University of Cambridge, Cambridge, CB2 7EF, United Kingdom

^ Should be considered as joint senior authors

*** Corresponding Author:** *Jason Stretton,* MRC Cognition and Brain Sciences Unit, 15 Chaucer Road, CB2 7EF, Cambridge, United Kingdom. [Jason.stretton@mrc-cbu.cam.ac.uk](mailto:Jason.stretton@mrc-cbu.cam.ac.uk)

# Supplementary Methods

## Additional information on clinical measures

*Structured Clinical Interview for DSM Axis-IV Disorders (SCID-I).*

The SCID-I is a standardized diagnostic interview schedule designed to assist clinicians and researchers in making reliable DSM-IV Axis I psychiatric diagnoses. The SCID-I involves a series of questions concerning current and past symptoms of a range of psychological disorders and usually takes between ½ and 1 hour. The SCID is only administered by experienced research staff that has undergone comprehensive SCID training. The mood module is used to verify whether participants are currently experiencing low mood of clinical severity or not. In this study, Depressed participants previously underwent a structural clinical interview (SCID-I) to confirm their diagnosis and current episode.

*Beck Depression Inventory (BDI-II).*

The BDI-II is a 21-item multiple-choice self-report measure assessing depressive symptomatology including low mood. It is one of the most widely used instruments for measuring the severity of depression with good internal consistency, test-retest reliability and convergent validity with standardized clinician assessments, enabling comparison across studies. The BDI-II is widely used as an assessment tool by health care professionals and researchers in a variety of settings. Scores on the BDI-II indicate any change in mood symptoms over time. Participants completed the Beck’s Depression Inventory (BDI-II) to assess current and residual symptoms of depression.

# Supplementary Behavioural Results

## Affective Ratings

### *Session I (Memory Generation Session) Results*

We investigated the composite affective ratings for inclusion, rejection and neutral social memories obtained within the initial Memory Generation session both for emotional impact upon recall and for retrospectively rated emotional impact at the time of the original event. The data are presented in Table S1. Mean composite affective ratings were entered into an ANOVA with group (Depressed/Healthy Controls) as a between-group factor and memory type (rejection/inclusion/neutral) and time (recall/experience) as the within-group factor. Within-subject factor memory type violated Mauchly’s test of sphericity (*x*^2^ = 13.26, p=0.001) and is reported using a Greenhouse-Geisser correction.

ANOVA results revealed a significant main effect for memory type (F[2,68]=521.43, p<0.001, η_p_^2^ =0.94). Planned comparisons indicated that mood ratings in response to rejection, neutral and inclusion memories all differed significantly from each other (all p<0.001). Mood following inclusion memories was significantly elevated relative to neutral memories (mean difference inclusion-neutral: 4.89 ± 0.37), and relative to rejection memories (mean difference inclusion-rejection: 13.61 ± 0.48). Mood following rejection memories significantly deteriorated relative to neutral memories (mean difference rejection-neutral: -8.72 ± 0.43), in line with predictions. There was also a main effect of time (F[1,34]=55.32, p<0.001, η_p_^2^=0.62), with significantly elevated mood ratings at time of recall compared to time of experience (mean difference: 1.25 ± 0.17), across groups. There was no main effect for group, although it approached significance (F[1,34]=4.14, p=0.05, η_p_^2^=0.11), revealing a trend for healthy Healthy Controls to show elevated mood relative to Depressed participants across memories. However, there was a significant interaction between memory type and time (F[2,68]=44.61, p<0.001, η_p_^2^=0.57) (see Figure S1). On closer inspection, this revealed that across both groups inclusion memories were rated as marginally less positive at time of recall compared to time of experience, while rejection memories were rated as significantly more positive at time of recall compared to time of experience. There was no significant interaction between memory type and group (F[2,68]=.81, p=.45, η_p_^2^=0.02), nor between time and group (F[2,68]=.69, p=.41, η_p_^2^=0.02). There was also no significant three-way interaction between time, memory and group (F[2,68]=.48, p=0.62, η_p_^2^=0.01).

Table S1. Mean Composite Affect Ratings from the Memory Generation Session

| \| Memory Type \| Time \| Group \| \| Mean \| \| Std. Deviation \| \| --- \| --- \| --- \| --- \| --- \| --- \| --- \| \| Neutral \| Experience \| \| Depressed \| \| 2.70 \| 1.96 \| \| Healthy Controls \| \| 2.81 \| 2.09 \| \| Total \| \| 2.75 \| 1.99 \| \| Recall \| \| Depressed \| \| 3.27 \| 1.94 \| \| Healthy Controls \| \| 3.26 \| 1.72 \| \| Total \| \| 3.27 \| 1.81 \| \| Rejection \| Experience \| \| Depressed \| \| -7.77 \| 1.54 \| \| Healthy Controls \| \| -7.03 \| 1.72 \| \| Total \| \| -7.42 \| 1.65 \| \| Recall \| \| Depressed \| \| -4.71 \| 2.53 \| \| Healthy Controls \| \| -3.32 \| 3.11 \| \| Total \| \| -4.05 \| 2.86 \| \| Inclusion \| Experience \| \| Depressed \| \| 7.61 \| 1.91 \| \| Healthy Controls \| \| 8.34 \| 1.07 \| \| Total \| \| 7.95 \| 1.59 \| \| Recall \| \| Depressed \| \| 7.32 \| 1.93 \| \| Healthy Controls \| \| 8.36 \| 1.32 \| \| Total \| \| 7.81 \| 1.73 \| |  |
| --- | --- | --- | --- | --- | --- | --- | --- | --- | --- | --- | --- | --- | --- | --- | --- | --- | --- | --- | --- | --- | --- | --- | --- | --- | --- | --- | --- | --- | --- | --- | --- | --- | --- | --- | --- | --- | --- | --- | --- | --- | --- | --- | --- | --- | --- | --- | --- | --- | --- | --- | --- | --- | --- | --- | --- | --- | --- | --- | --- | --- | --- | --- | --- | --- | --- | --- | --- | --- | --- | --- | --- | --- | --- | --- | --- | --- | --- | --- | --- | --- | --- | --- | --- | --- | --- | --- | --- | --- | --- | --- | --- | --- | --- | --- | --- |
|  |  |

**Figure S1. Mean ± 1SE of the Mean Composite Affect Ratings** from the Memory Generation Session reveals a significant interactive effect between time and memory type on mood in line with the established fading affect bias (Walker et al., 2003). Specifically, rejection memories were rated significantly less positive at time of experience relative to recall, compared to both inclusion and neutral memories, which were rated comparably in affect.

### *Session I (Memory-Generation Session) vs Session II (Neuroimaging Session)*

To ensure comparable saliency of memories across session, we then explored potential differences in affective experience in response to recalling personal memories within the initial Memory Generation session subsequent Neuroimaging Session, approximately one week later. Mean composite affective ratings at time of recall during each session were entered into an ANOVA with group (Depressed/Healthy Controls) as a between-group factor and memory type (rejection/inclusion/neutral) and time (session I/session II) as the within-group factor. See Table S2 for descriptive means. Both memory type (x^2^=10.60, p=0.005) and the interaction between memory type and time (x^2^=16.53, p<0.001) violated Mauchly’s assumption of sphericity and are reported using Greenhouse-Geisser.

The ANOVA results revealed a significant main effect for memory type (F[1.57,53.35]= 265.33, p<0.001, η_p_^2^=0.89). Planned comparisons of the main effect of memory type corrected using a Bonferroni adjustment, indicated that affective ratings (p<0.001) derived during rejection, neutral and inclusion memories differed significantly from each other, with greater positive mood in response to inclusion memories compared to neutral memories (mean difference 3.68±0.34) and inclusion compared to rejection memories (10.75 ±0.57). Rejection memories in turn elicited lower mood relative to neutral memories (-7.07±0.49). There was also a main effect of group (F[1,34]= 7.14, p=0.01, η_p_^2^=0.17), which in planned comparisons revealed significantly elevated mood in control participants compared to Depressed individuals (p=0.01) when collapsed across all memory types and time. Further, there was a significant interaction between time and memory type (F[1.44,48.78]=5.84, p=0.01, η_p_^2^=0.15) (see Figure S2), suggesting that, while overall there was comparable saliency across sessions and memory types, rejection in particular revealed a slight decrease in negative mood from session I to session II, while the reverse was observed for inclusion memories, which were rated as slightly less positive in the second session. However, importantly, there was no significant main effect for time (F[1,34]=0.01, p=0.94, η_p_^2^=0.00), no significant interaction between time and group (F[1,34]=0.39, p=0.54, η_p_^2^=.01), no significant interaction between memory type and group (F[2,34]=1.10, p=0.34, η_p_^2^=.03), and no significant three-way interaction between time, group and memory type (F[2,68]=0.46, p=0.64, η_p_^2^=.01) for affect rating across all memory types and sessions. Together, these results reassuringly suggest that memories elicited stable degrees of positive, negative and neutral affect as a function of memory type across research sessions. Similar patterns of stable findings were found for memory intensity and vividness ratings (Figures S3 and S4).

Table S2. Mean Composite Affect Ratings from the Memory Generation vs Neuroimaging Session

| Group | Memory Type | Session | Mean | Std. Error |
| --- | --- | --- | --- | --- |
| Depressed | Neutral | Session I | 3.02 | 0.44 |
|  |  | Session II | 3.13 | 0.58 |
|  | Rejection | Session I | -4.46 | 0.70 |
|  |  | Session II | -3.41 | 0.86 |
|  | Inclusion | Session I | 7.06 | 0.39 |
|  |  | Session II | 5.29 | 0.70 |
| Healthy Controls | Neutral | Session I | 3.49 | 0.42 |
|  |  | Session II | 4.20 | 0.55 |
|  | Rejection | Session I | -3.69 | 0.66 |
|  |  | Session II | -2.87 | 0.82 |
|  | Inclusion | Session I | 8.48 | 0.37 |
|  |  | Session II | 7.74 | 0.66 |

**Figure S2. Mean ± 1SE of the Mean Composite Affect Ratings** of present affective experience during the Memory Generation Session compared to present experience during the Neuroimaging Session revealed a significant interactive effect of memory type by time on mean composite affect ratings with inclusion memories rated more positively relative to neutral and rejection memories, across sessions.

**Figure S3. Mean ± 1SE of the Mean Composite Intensity Ratings** of present affective experience during the Memory Generation Session compared to present experience during the Neuroimaging Session.

**Figure S4. Mean ± 1SE of the Mean Composite Vividness Ratings** of present affective experience during the Memory Generation Session compared to present experience during the Neuroimaging Session.

### *Session II (Neuroimaging Session) Results*

Main results for the neuroimaging session affective results are presented in the main manuscript. However, to ensure that composite ratings of negative affect and positive affect scores did not mask underlying differences between groups as a function of individual affective ratings, repeated measures ANOVAs were carried out for each memory type and for each individual mood rating index (e.g. ‘Distress’, ‘Rejection’, ‘Inclusion’, ‘Positivity’) separately. See Figure S5 for overview of mean affective rating before and after each memory, presented for each group and memory type (Figure S5 A: Neutral Memory, B: Rejection Memory, C: Inclusion Memory).

In line with predictions, results revealed that for neutral memories, there was no significant main effect for time (F[1,35] = 0.84, p = 0.37, η² _p_ = 0.02), rating type (F[1,35] = 2.08, p = 0.16, η² _p_ = 0.06), or group (F[1,35] = 0.03, p = 0.86, η² _p_ = 0.001). This suggests that across rating indices (distress, rejection, inclusion, positivity) following neutral memories remained unchanged across groups.

For rejection memories, results revealed a significant main effect for time (F [1, 35] = 20.82, p < 0.001, η² _p_ = 0.34), and a significant interaction between group and rating type (F [3,105] = 102.79, p < 0.001, η² _p_ = 0.75). There was no significant main effect for rating type (p = 0.15), groups (p = 0.977) or no other significant interaction. This suggests that over time and across both groups, rejection memory lead to an increase in feelings of distress and rejection, alongside a decrease in positivity and sense of inclusion.

Finally, for inclusion memories, results revealed a similar pattern, with a significant main effect for time (F [1, 35] = 8.59, p = 0.006, η² _p_ = 0.20), rating type (F [3,105] = 53.65, p < 0.001, η²_p_ = 0.61) and a significant interaction between time and rating type (F [3,105] = 76.70, p < 0.001, η²_p_ = 0.69). There was no significant effect of group (p = 0.93) or other significant interaction. This finding suggests those following inclusion memories, positive mood and a sense of inclusion increased, while distress and feelings of rejection decreased.

Furthermore, repeated measures ANOVA revealed significant differences in the expected directions between the composite positive (F [1, 35] = 22.19, p < 0.001, η²_p_ = 0.37) and negative mood indices F [1, 35] = 46.04, p < 0.001, η²_p_ = 0.55) across both runs on mood scores recorded after the rejection memories and before the inclusion memories, indicating the successful effects of the washout clip.

There was also significant difference between the positive mood index (F [1, 35] = 43.69, p < 0.001, η²_p_ = 0.55) but not the negative mood index (F [1, 35] = 2.01, p = .16, η²_p_ = 0.05), recorded at the end of run 1 (post-positive memories) and beginning of run 2 (pre-neutral memories). This suggests the target manipulation of positive mood had returned to baseline prior to the second run while the negative mood index were unaffected. Overall, results suggest that composite affective ratings accurately describe the change in mood observed as a function of memory type.

# Supplementary fMRI Results

**Whole Brain ANCOVA; *Active Listening***

Whole-brain ANCOVA revealed a significant main effect of memory type was observed in the left inferior temporal gyrus and right middle occipital gyrus (See Table S3). Planned comparisons showed both clusters were more active during Inclusion memories, irrespective of group. There was no main effect of group or significant interaction between memory type and group.

**Table S3**. Whole Brain ANCOVA; Main effect of Memory Type during Active Listening

| Contrast | L/R | MNI Structural Atlas | MNI Coordinates (X,Y,Z) | k-voxels | z-score | p-value |  |  |
| --- | --- | --- | --- | --- | --- | --- | --- | --- |
| Main effect of Memory Type | | | | | | | |  |
|  | **L** | **Middle Temporal Gyrus** | **-52, -54, -14** | **546** | **5.17** | **0.000** |  |  |
|  | L | Middle Temporal Gyrus | -54, -58, 2 |  |  |  |  |  |
|  | L | Fusiform Gyrus | -44, -44 , 22 |  |  |  |  |  |
|  | **R** | **Middle Occipital Gyrus** | **44, -72, 24** | **237** | **5.08** | **0.000** |  |  |
| Abbreviations: R; Right, L; Left; p-value; FWE cluster level, initial height threshold uncorrected *p*<0.001 with an extent threshold of *k*=202. **Bold** font indicates peak co-ordinates of each cluster. | | | | | | | | |
